# Hospital Meetings

**Published:** 1896-03-21

**Authors:** 


### HOSPITAL MEETINGS.

### ROYAL HOSPITAL FOR DISEASES OF THE CHEST.—ANNUAL COURT OF GOVERNORS.

The eighty-second annual court of the Royal Hospital for Diseases of the Chest, City Road, was well attended on March 13th, the Right Hon. the Lord Mayor being in the chair. In proposing the adoption of the report of the council, with the balance-sheet, the Lord Mayor regretted to see a slight decrease in the annual subscriptions to the oldest hospital in Europe for diseases of the chest. He had been much pleased by the bright and comfortable wards] which he had inspected before coming to the meeting.

In the absence of Dr. Hensley, the senior physician, the resolution was seconded by Dr. Gilbart Smith, and carried. As a governor of the hospital, Dr. Gilbart Smith said he could speak from personal experience of what was done there. The report only furnished the merest outline of the work accomplished in the twelve months, but the outline could be easily filled in by those who knew how to do it. The improvements and cost of the drainage were visible facts, but there was also the battle with death and disease carried on incessantly in the wards. In this the physicians were materially aided by the admirable nursing staff. The science and medical art of to-day made work much more thorough and extensive than was the case twenty-one years ago, when Dr. Gilbart Smith said that he had first joined the hospital. The warfare carried on in the wards resulted in organised victory, for suffering was assuaged, lives saved, and death made easier to patients surrounded by comforts they could never enjoy at home.

A vote of thanks to the honorary officers was proposed by Mr. John S. Gilliat, M.P., who spoke of the good fortune of the institution in having as its president Lady Sarah Spencer, and as treasurer Lady Robert Bruce, both being always in the forefront of works of charity and good. Lord Robert Bruce's absence from the meeting on account of illness was deplored. The resolution was seconded by the Hon. Lionel Ashley (vice-chairman), who said that whatever views might be held with regard to the advantages of State-supported hospitals, whilst these institutions were dependent on charity voluntary assistance would always be necessary, and therefore it was fortunate that a certain number of ladies and gentlemen were found willing and able to give them their time and their money.

The Rev. W. H. Barlow, D.D., proposed a vote of thanks to the council and standing committees, and that the governors whose names were submitted should be elected to serve on the council. Dr. Barlow spoke of the advantage to the charity of having the names of men of integrity and repute associated with it, as they influenced others to subscribe; and, on the other hand, they did good to themselves by serving in philanthropic work. He thought that a very great debt of gratitude was due to the members of the council for their exertions.

In seconding the resolution, which was carried unanimously, Dr. Gilbart Smith referred to the excellent quality of the work and to the spirit of harmony and confidence which pervaded the institution. The efforts of the medical officers were aided and abetted by men of experience who were no novices in the art of hospital management. The sick were no longer left to the Samaritan, for priest and Levite were now continually serving them.

Mr. T. Andros de la Rue moved a vote of thanks to the honorary medical officers, and spoke in high terms of the kindness as well as skill which the patients received. He did not think there was a hospital in London where the council and medical officers worked so harmoniously together.

The vote, which was carried unanimously, was seconded by the Rev. J. H. Rose, who said he did not know why doctors should be expected to give their services freely. They had gone through a long and expensive education, and yet it was taken as a matter of right that they, unlike other men, should work for nothing.

Dr. Oswald Browne, in returning thanks for the medical staff, spoke of the general goodwill exhibited towards the hospital, which was undoubtedly very popular with the poor of the district.

Colonel Makins (trustee) moved a vote of thanks to the Lord Mayor for his kindness in presiding. The charities always received recognition at the hands of the Chief Magistrate. He might say that the Lord Mayor was the master turncock of the stream of public charity. An outburst of patriotism was appreciated, and there might also well be an outburst of charity.

Mr. T. Andros de la Rue seconded the resolution, which was carried unanimously.

The Lord Mayor briefly responded, and the hospital was afterwards inspected by the ladies and gentlemen present at the meeting.

### DENTAL HOSPITAL OF LONDON.—ANNUAL GENERAL MEETING.

The annual general meeting of the Dental Hospital of London, Leicester Square, was held on March 12th. Mr. F. A. Bevan was in the chair, and proposed, as the first resolution, the adoption of the annual report. He referred to his father having been treasurer to the hospital, and said he felt that he had inherited from him his own love for it. The premises were unfortunately now totally inadequate; in fact, the work had completely outgrown the present accommodation, and it was absolutely necessary to get into fresh quarters. A site had been secured, and considerable progress made towards the desired end, and this was chiefly owing to the zeal of their treasurer, Dr. Walker. The Goldsmiths' Company had sent the hospital a donation of £100, and if the other City companies would do the same there would be no longer any lack of support. Mr. Bevan said that hardly a day passed without his receiving applications for letters for the Dental Hospital, which showed a wide-spread appreciation of its work. If all those who benefited by the institution would take the trouble to make known its good work, better support would be forthcoming. Everybody suffered from their teeth at one time or other, therefore all should sympathise with the excellent work accomplished by the Dental Hospital. He believed if people would but come and see the inconvenience and crowded condition of the present premises they would be glad to help to improve matters.

Mr. David Hepburn seconded the adoption of the report, and believed that publicity would greatly aid in opening the coffers of those who could well afford to assist the hospital.

After the adoption of the report, a resolution for the re-election of members was moved by Mr. Hutchinson, seconded by Mr. Morton Smale, secretary, and carried.

The election of new members was proposed by Mr. W. H. Ash, seconded by Mr. Storer Bennett, and carried.

Mr. Alfred Marsh moved the re-election of the treasurer, Dr. Walker, whose name was received with great applause; Mr. W. H. Ash seconded; this was carried unanimously.

Dr. Walker acknowledged the compliment paid him, and said that the responsibilities of trustee and treasurer to the hospital had caused him many anxious and sleepless nights, but he was willing to carry on the duties until a younger and more able man was found to succeed him.

The re-election of the auditors was proposed by Mr. Canton, seconded by Mr. W. C. Smale, and carried.

In proposing the vote of thanks to the treasurer, chairman, committee of management, and medical officers, Mr. David Hepburn said that their present satisfactory condition was largely due to Mr. Stoneham, chairman of the Managing Committee, whose absence from the meeting on account of illness was a source of regret. Mr. Smith Turner, the deputy chairman, was also a tower of strength. Mr. Hepburn considered that the lay element was of great value on the committee, and brought enthusiasm and valuable assistance to the financial and administrative departments. The monthly balance-sheet, prepared with so much care and patience by these gentlemen, was greatly appreciated. Neither the public nor even the committee of management could realise the disadvantageous circumstances amidst which the medical staff had to carry on their work in the present overcrowded and unsuitable premises.

This vote of thanks was seconded by Mr. E. LLOYD WILLIAMS, and carried.

The TREASURER spoke of the excellent assistance given by the Finance Committee and of the unceasing kindness and help which he had received from Mr. Bevan at all times, and he proposed a vote of thanks to him for taking the chair.

Mr. SMITH TURNER, in seconding the vote, remarked that it was a matter of surprise that the public did not afford more support. He, and doubtless others, received constant applications for letters from people who wanted them "to give away," and who seemed to think they cost nothing; and he must say that many who thus applied were perfectly able to subscribe and obtain letters for themselves. He pointed out that, although the work of the hospital had so enormously increased in the matter of stoppings, &c., extractions had diminished, and forceps were no longer the chief instruments in a dentist's store, as was formerly the case.

The CHAIRMAN returned thanks to the meeting, and considered that there had been a good and cheerful tone about the proceedings. He was glad to note that teeth were kept in people's heads.

### NATIONAL SOCIETY FOR EMPLOYMENT OF EPILEPTICS.

The annual meeting of governors was held at the offices, 12, Buckingham Street, on Monday, March 9th, the chair being taken by Mr. E. MONTEFIORE MICHOLLS. In the report presented by the hon. medical staff it is stated that "the experience of the past twelve months has entirely justified the sanguine expectations expressed in the report of last year that the epileptic colony" (at Chalfont St. Peter) "has now successfully passed the experimental stage, and the provision of suitable employment as a form of remedial treatment has been attended with the happiest results." The executive committee plead for the numerous and daily-increasing body of disappointed applicants whose hopes of entering the colony can never be realised until funds are provided for increasing the accommodation, which is at present totally inadequate to the demand.

The CHAIRMAN remarked that cases were admitted impartially to the colony, so far as there was room for them, from all parts of Great Britain, but the support given to the society came mainly from London. The committee hoped that residents in provincial towns willing to co-operate would communicate with the secretary in order that measures might be taken for arousing interest in the movement in every town and village throughout the country. He (the chairman) looked forward to the time when there would be in England not one national epileptic colony but several colonies, under local management, but until that time came the society felt that, taking cases so widely as they did, they might fairly expect more support from the provinces than had yet been received.

There are at present 36 colonists at Chalfont St. Peter, and a new home for 24 women is to be at once erected through the generosity of Mr. Passmore Edwards, who has undertaken to defray the whole expense, and has, moreover, stated that this offer will not affect his previous intention to build two homes for children, though these will not be begun until after the completion of the women's home. Amongst immediate necessities mentioned in the report is the establishment of a house for a resident medical officer, and it is suggested that if a suitable house can be built with room for a certain number of private patients, the payments of the latter would go towards the general support of the colony. "There is," continues the report, "no way in which friends of the society could better help us than by contributing towards the cost of erecting such a house."

### BIRMINGHAM AND MIDLAND HOSPITAL FOR WOMEN.

There was a well-attended annual meeting of governors of this hospital on Wednesday, March 11th, the Countess of Dudley, president of the hospital, being in the chair. Amongst those present were Dr. Savage, Messrs. G. Hookham, P. Hookham, and Lawson Tait. The report shows that the number of new cases treated in the out-patient department during the year 1895 was 2,925, an increase of 22 compared with the previous year, the total number of new and old attendances during the year being 17,563. The number of in-patients admitted during the year was 359, an increase of eight over the previous year. During 1895 special efforts have been made to increase the subscription list, and the personal efforts of the committee in this direction have been supplemented by the employment of two paid canvassers during considerable portions of the year, and a third canvasser has recently been appointed for the outlying districts round Birmingham. The result of these special efforts has been the addition of a little over £200 to the subscription list up to the present time, an increase of over 50 per cent. The sale of work held in November realised the satisfactory sum of £430, of which £230 went to pay off the debt on the building account, £100 to the convalescent fund, and £100 to the general funds. Despite these additions, however, the finances of the hospital are very much straitened, and in but little better condition than at the end of 1894. It is, therefore, hoped that the public will generously come forward with increased support, so that the valuable work of this hospital may not be curtailed, it being impossible for it to continue on the present financial basis. In accordance with a suggestion made at the last annual meeting, the committee have made a new regulation to the effect that any person whose usual place of residence is more than twenty-five miles from Birmingham will now only be admitted to the hospital on payment of a sum of £4, which amount has been found to be about the average cost per patient to the hospital.

### ROYAL HOSPITAL, RICHMOND.

Sir J. WHITTAKER ELLIS presided at the recent annual meeting of the governors and subscribers of the Richmond Royal Hospital in the unavoidable absence of the Duke of Teck. The report before the meeting announced that the new buildings, the Princess May's Ward for children and an isolation ward, were nearly completed, and that it was hoped a public opening would take place during the summer. The report drew attention to the fact, emphasised later on by Colonel Sparks and other speakers, that although the past year had brought to the hospital several important legacies, resulting in a balance in the hands of the trustees of £338 16s. 4d., yet these very legacies have been due to the deaths of several good friends to the institution, and that the annual subscriptions had been steadily decreasing of late years, a very serious fact when it was remembered that the opening of the new children's ward would mean a considerable rise in yearly expenditure.

At a special meeting held subsequently, it was decided to expunge a bye-law whereby the cost of maintenance of in-patients who were domestic servants was charged to their masters or mistresses. It was a rule which was found almost unworkable in practice, because some subscribers of two or three guineas a week resented a charge being made, and had even withdrawn their subscriptions in consequence, and because people of small means practically discharged their servants before they were received into the hospital to avoid the expense. It was finally unanimously decided to do away with the rule, Mr. MAX WAECHTER remarking that he thought if masters and mistresses were made aware that a donation would be expected from them, they would gladly fall in with the suggestion.

Since this meeting took place the secretary of the Royal Hospital has received a cheque for twenty guineas from Mr. Waechter, and an intimation that he will pay that sum annually to cover any deficiency in revenue resulting from the decision of the governors to abandon payment for domestic servants.

